# Metformin reduces glucose intolerance caused by rapamycin treatment in genetically heterogeneous female mice

**DOI:** 10.18632/aging.101401

**Published:** 2018-03-22

**Authors:** Roxanne Weiss, Elizabeth Fernandez, Yuhong Liu, Randy Strong, Adam B. Salmon

**Affiliations:** 1 Geriatric Research, Education and Clinical Center, South Texas Veterans Health Care System, San Antonio TX 78294, USA; 2 The Sam and Ann Barshop Institute for Longevity and Aging Studies, The University of Texas Health Science Center at San Antonio, San Antonio TX 78229, USA; 3 Department of Pharmacology, The University of Texas Health Science Center at San Antonio, San Antonio TX 78229, USA; 4 Department of Molecular Medicine, The University of Texas Health Science Center at San Antonio, San Antonio TX 78229, USA

**Keywords:** mTOR, gluconeogenesis, AMPK, leptin, adiponectin, interventions, insulin

## Abstract

The use of rapamycin to extend lifespan and delay age-related disease in mice is well-established despite its potential to impair glucose metabolism which is driven partially due to increased hepatic gluconeogenesis. We tested whether a combination therapeutic approach using rapamycin and metformin could diminish some of the known metabolic defects caused by rapamycin treatment in mice. In genetically heterogeneous HET3 mice, we found that chronic administration of encapsulated rapamycin by diet caused a measurable defect in glucose metabolism in both male and female mice as early as 1 month after treatment. In female mice, this defect was alleviated over time by simultaneous treatment with metformin, also by diet, such that females treated with both drugs where indistinguishable from control mice during glucose tolerance tests. While rapamycin-mediated glucose intolerance was unaffected by metformin in males, we found metformin prevented rapamycin-mediated reduction in insulin and leptin concentrations following 9 months of co-treatment. Recently, the Interventions Testing Program showed that mice treated with metformin and rapamycin live at least as long as those treated with rapamycin alone. Together, our data provide compelling evidence that the pro-longevity effects of rapamycin can be uncoupled from its detrimental effects on metabolism through combined therapeutic approaches.

## Introduction

Pharmaceutical inhibition of the mechanistic target of rapamycin (mTOR) is now well established as a valuable tool to better understand ways by which the aging process can be delayed in mammals. In multiple studies, chronic treatment with the mTOR inhibitor rapamycin significantly extends mouse lifespan in both males and females even when begun late in life [1-5]. Moreover, rapamycin delays the progression of several age-related pathologies and may be capable of revitalizing age-related declines in some tissues [5-9]. At the same time, chronic administration of rapamycin is known to have side-effects such as increased incidence of metabolic dysfunction, including glucose intolerance and insulin resistance, likely hindering its use in human populations as an anti-aging treatment. There is emerging evidence that these metabolic defects caused by rapamycin might be alleviated by adjusting treatment protocols, including use of an intermittent treatment schedule or drug-free holidays [10-12]. It is still unclear whether these alternative treatment regimens fully recapitulate the effects of chronic rapamycin on longevity or healthy aging, though there is growing evidence that at least some of these effects are likely to be maintained [13-15],

Another alternative approach may be to utilize a combination of therapeutics in which the side-effects of chronic rapamycin are treated simultaneously with an additional pharmaceutical. In regards to metabolism, co-treatment with rosiglitazone, a PPARγ ligand and insulin sensitizer, has been shown to partially rescue glucose intolerance and insulin resistance in rapamycin-treated rats [16]. While rosiglitazone is an effective insulin sensitizer, evidence suggests that rapamycin-induced glucose intolerance is mediated through increased hepatic gluconeogenesis [17-19]. Metformin is part of the first-line treatment for type 2 diabetes mellitus and reduces hyperglycemia primarily by inhibiting hepatic gluconeogenesis [20,21]. Independent of this outcome, metformin has also been shown to increase peripheral insulin sensitivity [22], increase peripheral glucose uptake [23], increase fatty acid oxidation [24] and decrease absorption of glucose from the digestive system [25]. The mechanism of action for metformin is still controversial and metformin has been proposed to stimulate the AMP-activated protein kinase (AMPK) pathway by inhibiting complex I activity in the mitochondria [26,27], by inhibition of mitochondrial redox shuttle enzymes [28] or through mitochondria independent mechanisms [29]. Interestingly, metformin also inhibits mTORC1 signaling through S6K1 and 4E‐BP1 and results in decreased protein translation [30]. In contrast to rosiglitazone, metformin is relatively safe, has been used extensively in the US for more than 20 years and its long-term effects in humans have been well-studied [31].

As part of the Interventions Testing Program (ITP), the 2011 longevity cohort tested the effect of combined metformin and rapamycin treatment on the lifespan of HET3 mice [32]. In these recently published data, both female and males in this combined treatment group lived 23% longer than control mice suggesting that combining metformin with rapamycin results in a lifespan extension at least as great as (if not greater than) rapamycin treatment alone. As an extension of these findings, we used this experimental paradigm to test what effect chronic treatment with both pharmaceuticals has on metabolic function of mice. We report here that metformin can almost completely abolish glucose intolerance in female HET3 mice treated with rapamycin. In male HET3 mice treated with rapamycin, glucose intolerance is unaffected by metformin treatment; however, metformin does reverse the effect of rapamycin on several metabolic hormones. These data then provide compelling evidence that the pro-longevity effects of rapamycin can be uncoupled from its detrimental effects on metabolism through combined therapeutic approaches.

## RESULTS

Four month old male and female HET3 mice were randomly assigned to one of four groups fed the following diets based on LabDiet 5LG6: diet only (Control), 14 ppm encapsulated rapamycin (eRapa), 0.1% metformin (Met), or both 14 ppm encapsulated rapamycin and 0.1% metformin (eRapa+Met). For the duration of the experiment, mice were given access to diet and water ad libitum.

### Body weight and composition

**Figure 1:**
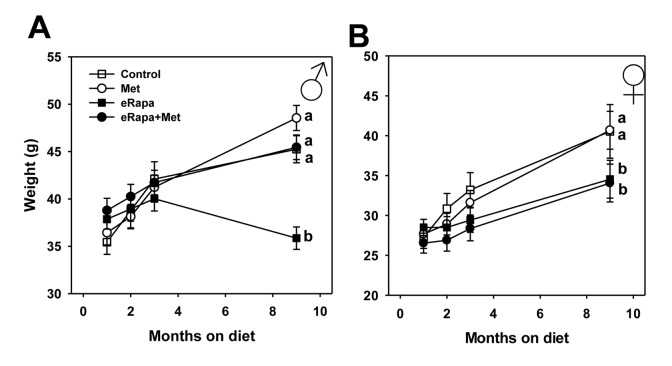
**Metformin prevents rapamycin-induced weight loss in male mice.** Body weights over time for (**A**) male and (**B**) female HET3 mice fed control (open square) diet or diets containing metformin (open circle), rapamycin (closed square), or both metformin and rapamycin (closed circle). Symbols represent mean values at indicated time point ± SEM. For all groups, n=10. Letters indicate significant difference among groups.

In male mice, the four groups did not differ in weight through 3 months of treatment. After 9 months of treatment, eRapa males weighed significantly less than all other groups including eRapa+Met mice suggesting that metformin abolishes this effect of rapamycin. Females treated with rapamycin, either eRapa or eRapa+Met, weighed significantly less than control and Met-treated females starting at 3 months of treatment and continuing through 9 months (Figure 1). The masses of most fat depots were reduced in mice fed rapamycin at this 9 month point, as were hind-limb skeletal muscle mass in males and kidney mass reduced in female mice fed rapamycin (Supplementary Table 1). Metformin had no effect on tissue mass at this time point except for a small reduction in hind limb muscle mass in females.

### Alterations in glucose metabolism

**Figure 2:**
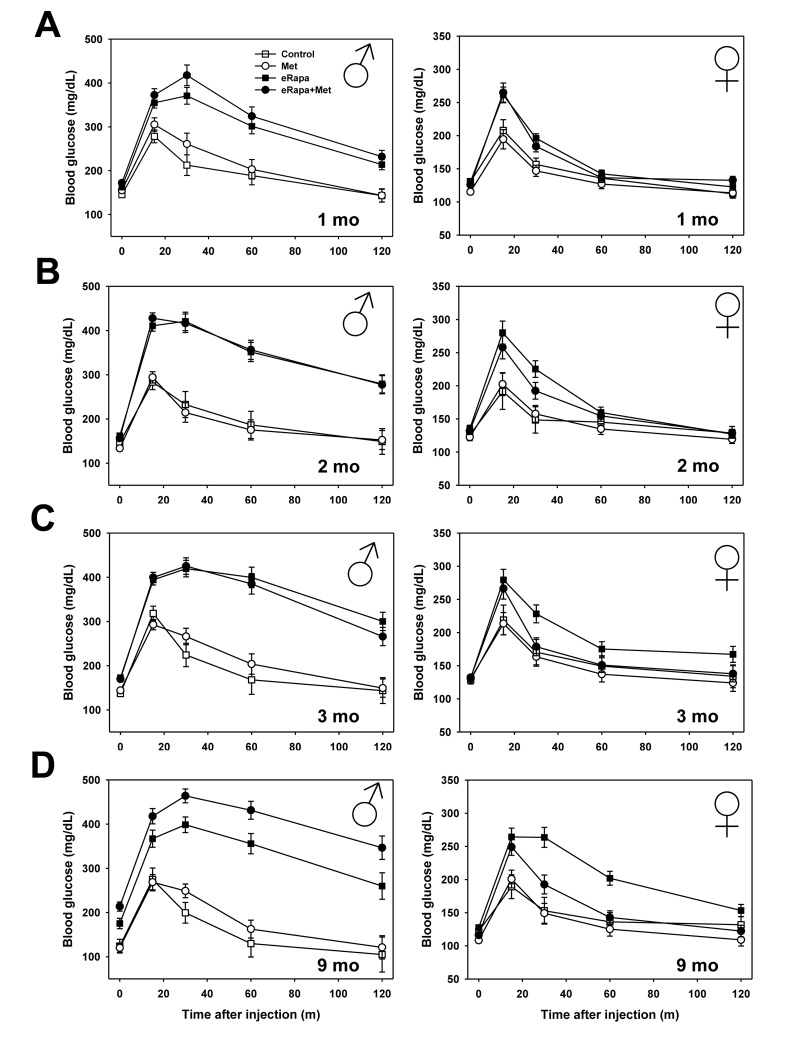
**Metformin abrogates rapamycin-mediated glucose intolerance in female mice.** Glucose tolerance tests performed in male (left) and female (right) mice following (**A**) 1, (**B**) 2, (**C**) 3, or (**D**) 9 months of indicated diet treatments. Symbols represent mean values for indicated group at each time point ± SEM. For all groups, n=8-10.

**Figure 3:**
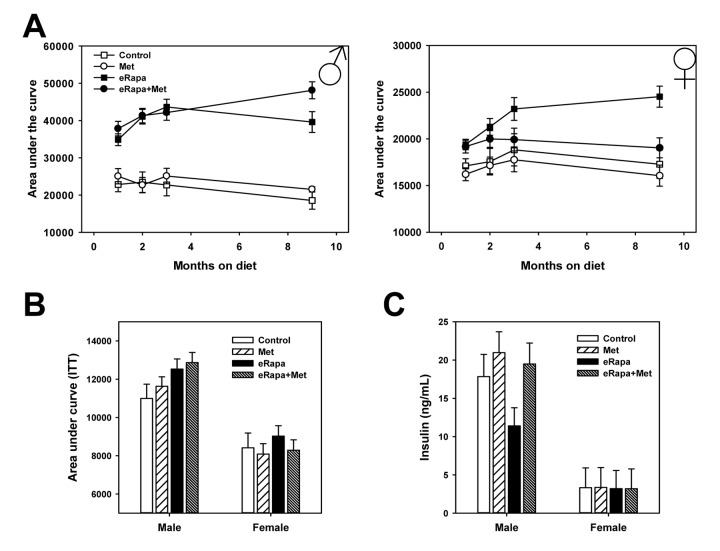
**Combined effects of rapamycin and metformin on glucose metabolism.** (**A**) Area under the curve for glucose tolerance tests repeated in the same cohort of animals following indicated months of treatment. Symbols represent mean values for indicated group at each time point ± SEM. (**B**) Area under the curve calculated for insulin tolerance tests performed following 3 months of treatment. Bars represent mean values for indicated group at each time point ± SEM. (**C**) Insulin concentration in plasma collected from fed mice following 9 months of treatment on the indicated diets. Bars represent mean values for indicated group at each time point ± SEM. For glucose and insulin tolerance test, n=8-10 for all groups. For insulin measurements, n=8-10 for all groups.

The primary goal of this study was to determine whether metformin could improve the reported defects in glucose metabolism that are caused by short-term and chronic treatment with rapamycin. For both males and females, metformin alone had no effect on glucose tolerance compared to controls at any point during this study. Martin-Montalvo et al. showed a similar outcome in male C57BL/6 mice using the same 0.1% metformin dose [33]. In contrast, both males and females, eRapa treatment caused significant glucose intolerance beginning at our initial assessment point 1 month after treatment. In both sexes, metformin had no effect on glucose tolerance either alone or as an interaction with rapamycin (Figure 2 and Supplementary Table 2). In the same animals, we repeated glucose tolerance tests at 2, 3 and 9 months after treatment was begun (Figure 2 and Figure 3A). In males, rapamycin had a significant effect on the area under curve (AUC) calculated for glucose tolerance tests at all times tested. Metformin had no significant effect either alone or as interaction with rapamycin (Supplementary Table 2).

In females, rapamycin had a significant effect on glucose tolerance at all points tested. Metformin had no significant effect through 3 months of treatment, but did have a significant effect at 9 months of treatment. Ad hoc multiple comparison (Holm-Sidak) testing of female treatment groups found a significant effect of metformin among those mice treated with rapamycin (p=0.001) but no effect of metformin within mice not treated with rapamycin (p=0.52). Thus, metformin at this dose reduces the glucose metabolic dysfunction caused by rapamycin in female, but not male, HET3 mice.

In males, rapamycin increased fasting blood glucose concentrations as early as 1 month on the diet and continued throughout the duration of the experiment (Supplementary Table 3). Metformin had little effect on male fasting glucose at the times measured. In contrast, rapamycin had no effect on fasting blood glucose in female mice throughout the study. Further, metformin reduced female blood glucose concentrations both 1 month and 9 months after treatment was begun. Using insulin tolerance tests, we found that rapamycin causes mild insulin resistance in male mice whether given alone or in conjunction with metformin (Two way ANOVA eRAPA F=5.7, p=0.025; Met F=0.7, p=0.41, interaction F=0.1, p=0.81). In females, neither rapamycin nor metformin had any effect on insulin sensitivity as measured by this test (Figure 3B and Supplementary Figure 1).

### Endocrine regulation and hepatic gluconeogenesis

**Figure 4:**
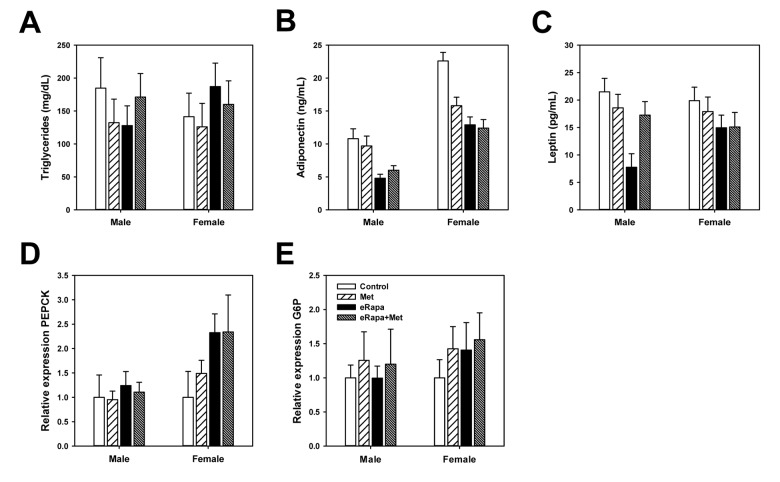
**Rapamycin and metformin effects on circulating metabolic markers.** (**A**) Triglycerides, (**B**) Adiponectin and (**C**) Leptin in plasma collected from fed mice following 9 months of treatment on the indicated diets. For A-C, n=8-10 for all groups. Rapamycin and metformin effects on hepatic gluconeogenesis. (**D**) relative phosphoenolpyruvate carboxykinase (PEPCK) expression and (**E)** relative glucose 6-phosphatase (G6P) expression in liver from male and female mice fed indicated diets. For D and E, n = 5 for all groups. For all, bars represent mean values for indicated group ± SEM.

In the interest of identifying the effect of chronic rapamycin and metformin under normal conditions, tissues including plasma were collected from animals in the ad libitum fed rather than fasted conditions. In males, there was no significant effect of rapamycin or metformin on insulin concentrations when analyzed by 2 way ANOVA (Figure 3C). However, when analyzed by ANOVA (*i.e.*, each treatment group as an independent variable) the eRapa treated mice had significantly reduced insulin concentration compared to control (Holm-Sidak multiple comparison p = 0.024). Neither metformin nor rapamycin had any effect on insulin concentrations in female mice. Interestingly, insulin concentrations in females were ~1/5 of that of males corresponding to the relative insulin sensitivity of female HET3 mice compared to males. Plasma triglycerides in both male and female mice were unaffected by treatment (Figure 4A).

Adiponectin and leptin are two hormones released by adipose tissue that play key roles in the regulation of glucose metabolism. In males, rapamycin significantly reduced plasma concentrations of adiponectin with no effect of metformin (2 way ANOVA eRAPA F=45.8; p<0.001; metformin F=0.02, p=0.8; interaction F=2.4, p=0.13). Rapamycin also significantly reduced leptin concentrations in males which appeared to be restored by concurrent metformin treatment (2 way ANOVA eRAPA F=13.7, p=0.001; metformin F=2.6, p=0.12; interaction F=9.3, p=0.05) (Figure 4B). In females, both rapamycin and metformin reduced adiponectin concentrations with no significant interaction (2 way ANOVA eRAPA F=16.5, p<0.001; metformin F=5.1, p=0.03; interaction F=3.7, p=0.06). Leptin concentrations in females were unaffected by either metformin or rapamycin (Figure 4C).

Previous studies suggest rapamycin-induced impairments in glucose metabolism are largely due to hepatic insulin resistance and failure of suppression of hepatic gluconeogenesis [17,34]. Gene expression of gluconeogenesis regulators phosphoenolpyruvate carboxykinase (PEPCK) and glucose 6-phosphatase (G6P) were unchanged by treatment in males (Figures 4D and 4E**)**. G6P was unaffected by either treatment in females, but rapamycin increased expression of PEPCK in females with no effect of metformin (2 way ANOVA eRAPA F=4.3, p=0.05; metformin F=0.2, p=0.65; interaction F=0.2, p=0.65).

### mTOR and AMPK signaling

**Figure 5:**
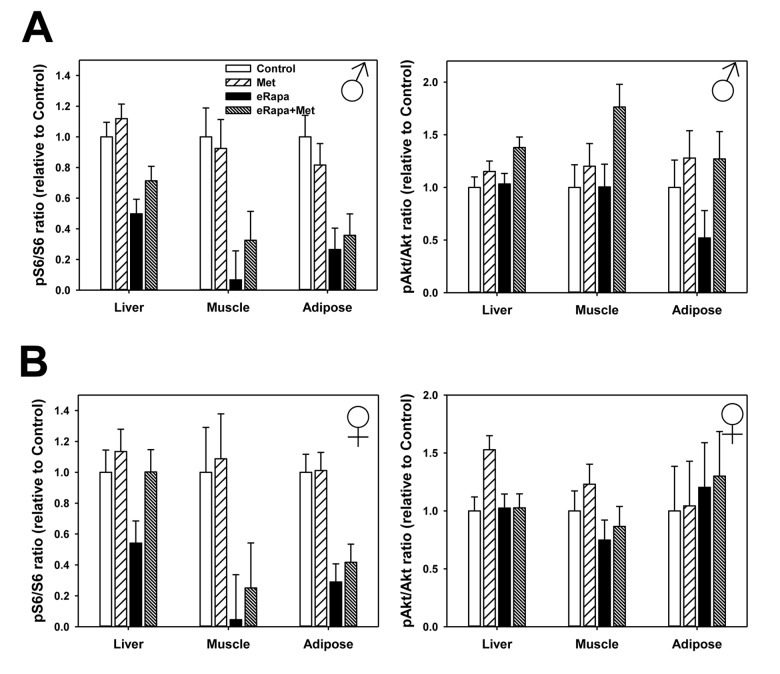
**No effect of metformin on rapamycin-mediated mTORC1 inhibition.** Quantification of phosphorylation/total protein ratios for S6 (left) and Akt (right) for liver, adipose tissue and muscle collected from male (**A**) and female (**B**) mice. Bars represent mean values for indicated diet/sex ± SEM. For all groups, n=6.

In males, rapamycin generally reduced mTORC1 signaling (as measured by p-S6) in liver, skeletal muscle and adipose tissue (Figure 5, Supplementary Figure 2 and Supplementary Table 4). Metformin had no significant effect nor was there an interaction effect in males suggesting this drug did not alter S6 phosphorylation or the effect of rapamycin. In females, rapamycin significantly reduced mTORC1 in skeletal muscle and adipose and similar to males there was no effect of metformin or interaction in these tissues. In female liver, we found no significant effect of rapamycin on phosphorylation of S6 when analyzed by 2 way ANOVA. However, when only control females and the eRapa females were compared by t-test, there was a significant (p=0.025) reduction in S6 phosphorylation and no significant difference between control and eRapa+Met groups.

We found that rapamycin had much less effect on mTORC2 signaling (as measured by Ser473 phosphorylation of Akt) in both sexes with the only significant effect on a small increase in female adipose. Metformin also had little effect on mTORC2 with the only significant effect a small increase in male liver (Figure 5, Supplementary Figure 2 and Supplementary Table 5).

**Figure 6:**
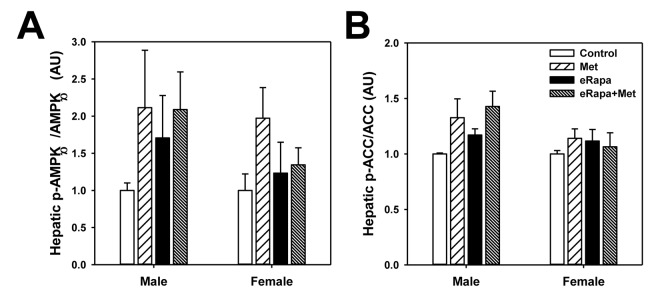
**Mild activation of AMPK signaling in metformin treated mice.** Quantification of phosphorylation/total protein ratios for (**A**) AMPKα and (**B**) ACC from liver of male and female mice fed indicated diets. Bars represent mean values for diet/sex ± SEM. For all groups, n=6.

While the mechanism action of metformin is incompletely understood, it has been reasonably well-accepted that metformin activates the AMP-activated protein kinase (AMPK) signaling pathway particularly in the liver. In male mice, phosphorylation of the AMPKα subunit was unchanged by either rapamycin or metformin (2 way ANOVA eRAPA F=0.4, p=0.54, metformin F=1.9, p=0.19, interaction F=0.4, p=0.51) (Figure 6 and Supplementary Figure 2). We found a similar results for AMPKα subunit phosphorylation in females (2 way ANOVA eRAPA F=0.4, p=0.57, metformin F=2.6, p=0.13, interaction F=1.7, p=0.22). However, metformin significantly increased phosphorylation of one of the effectors of AMPK, Acetyl-CoA carboxylase (ACC), in male (2 way ANOVA eRAPA F=1.4, p=0.25, metformin F=6.7, p=0.024, interaction F=0.1, p=0.76), but not female mice. In general, these data point to only moderate activation of AMPK signaling at this dose of metformin delivered through the diet.

One potential reason for the differences between males and females in the outcomes we report above might be sex-dependent differences in metabolism of rapamycin [1]. Unlike previous reports using this encapsulated version of rapamycin, we found no difference in blood rapamycin concentration between male and female mice treated with rapamycin alone (Table 1). Surprisingly, the combination of metformin and rapamycin resulted in significantly reduced blood concentrations of rapamycin in females though not males. While this could partially explain the reduced mTOR inhibition in female liver, it is not clear why we did not also not similar decreases in mTOR signaling in female muscle and adipose. Perhaps even more surprisingly, the concentration of metformin was significantly reduced in animals treated with both drugs in comparison to those treated with metformin alone. This occurred in both male and female mice.

**Table 1:** Blood concentrations of rapamycin and metformin at sacrifice.

**Group**	**Rapamycin [ng/mL]**	**p value:** eRapa vs eRapa+Met	**Metformin [µg/mL]**	**p value:** Met vs eRapa+Met
*Male*				
eRapa	38 ± 7		-	
Met	-		2.1 ± 0.3	
eRapa+Met	41 ± 7	0.79	1.3 ± 0.2	***0.04***
				
*Female*				
eRapa	43 ± 8		-	
Met	-		3.9 ± 1.0	
eRapa+Met	27 ± 4	*0.07*	1.3 ± 0.2	*0.07*
				

p value presented is for t test comparing values of indicated groups. Dash indicates levels of this drug in indicated group fall below level of detection. Control groups for both males and females have undetectable levels of both rapamycin and metformin.

## DISCUSSION

Our main finding here is that a multi-drug approach utilizing metformin can alleviate common metabolic deficits associated with chronic rapamycin treatment as a pro-longevity therapeutic. Moreover our data are in line with recent studies suggesting the metabolic dysfunction of rapamycin can be dissociated from its molecular effects on inhibition of mTOR either pharmaceutically or through alternative pharmaceutical regimens [10,16]. This has been an ambiguous question since the initial report of the pro-longevity effect of rapamycin administration in mice by the ITP in 2009 [1]. One interpretation of this seemingly paradoxical outcome is that rapamycin promotes long life despite also developing glucose intolerance, insulin resistance and/or decline in insulin production and that preventing these metabolic defects may extend lifespan further still. While glucose impairments promote clear health deficits in humans, one potential confound of this interpretation for mouse studies is that their lifespan is typically thought to be due to the development of cancer rather glucose metabolic dysfunction. However, such metabolic impairments have been associated with the acceleration of cancer progression and even the development of certain types of cancer in mouse models suggesting that glucose metabolism could impact mouse mortality at least indirectly [35,36]. The ITP has recently reported the lifespan of eRapa+Met mice is at least as long, if not longer than historical data for mice treated with eRapa alone [32]. Together, our data provide modest evidence for this interpretation, but leave open questions regarding the next steps in research pursuit. While both young and old mice are susceptible to rapamycin-mediated glucose intolerance [34], it is unknown whether metformin would have the same benefit in aged mice. More testing will be required to address whether metformin can alleviate the greater degree of glucose intolerance caused by higher doses of rapamycin than the 14 ppm dose used here [37].

The effects of rapamycin on glucose intolerance have been largely attributed to the inhibition of mTORC2, rather than mTORC1, that occurs with chronic administration of rapamycin [17]. Genetic ablation of mTORC2 in mice is sufficient to cause glucose intolerance in both sexes and shorten lifespan in male, but not females [18]. Beyond glucose intolerance in our mice, we found only relatively minor effects of rapamycin on markers of gluconeogenesis. One possibility for these small effects may be the relative lack of mTORC2 inhibition we noted in our rapamycin-treated mice. The effect of rapamycin on mTORC2 does seem to be heavily dependent on several factors including tissue type, genetic background, diet, fed or fasted state [11,18,38]. Our data show strong effects of rapamycin on mTORC1 signaling after 9 months in liver, muscle and adipose but relatively little effect of rapamycin (or metformin) on mTORC2. However, the tissues we used were collected from mice in a time restricted fed, rather than fasted, state. Schreiber et al. recently showed that harvesting conditions, particularly whether mice were insulin injected or not, can dramatically alter the outcome of rapamycin-mediated mTORC2 inhibition *in vivo* [38]. Moreover, a careful examination of potential compensatory effects on mTORC2 signaling due to such long-term (9 months) treatment with chronic rapamycin have not yet been performed.

Interestingly, we show several disparities between male and female mice in terms of both rapamycin as well as combined rapamycin and metformin treatment. It has been well-established that rapamycin extends lifespan to a greater degree in female compared to male mice [1,2,4,37]. It is not clear here why only females benefited from metformin treatment in regards to rapamycin-mediated glucose intolerance, however it seems likely to be related to reduced severity of glucose dysfunction in female mice treated with rapamycin compared to males [37,39]. Here, rapamycin did not increase fasting blood glucose in female mice at any time and glucose intolerance in female mice was approximately two-fold less than that caused in male mice. Interestingly, metformin did not immediately alleviate the effects of rapamycin on glucose intolerance. As Figure 3A shows, rapamycin-treated mice became progressively more glucose intolerant over the course of 9 months; metformin showed no effect early but cumulative use of metformin seemed to blocks this progression. Short-term treatment with metformin is known to have some beneficial effects, though the long-term benefits, including potentially lifespan extension, are associated with cumulative use [40,41]. Our study was limited in sample availability, so an important future study should address the molecular and physiological events at each of the time points (or more) used in our study. In previous mouse studies, it has been reported that metformin can preferentially reduce cancer and extend longevity in female but not male mice [42]. However, it is of interest that we also noted male-specific effects of combined metformin and rapamycin treatment. Rapamycin-mediated changes in body weight and insulin concentration all seemed to be altered by metformin in males but not females. The changes in leptin associated with the combined treatment in male mice is likely related to the difference in body weight, though further study would be required to delineate this relationship. It is unclear on why metformin also seems to counter the loss of weight caused by rapamycin in male mice. While we did not collect food consumption data, it is possible that cell non-autonomous mechanisms, in the brain for example, that regulate food intake could be affected by both drugs. Again, it is unclear why this would preferentially affect male and not female mice in this study. It would be of interest to test whether longer treatments or higher doses of metformin might also alleviate glucose intolerance in male mice. However, it must be noted that a metformin dose 10-fold higher than used here has been reported as toxic to mice [33].

Perhaps most interesting in this study is the finding that combined treatment with metformin and rapamycin reduces the effective concentration of rapamycin (in females) and metformin (in both sexes) relative to treatment with either drug alone. While mathematically the diet which contains both drugs would have less of each drug available on a per gram basis, this difference is relatively tiny and unlikely to produce such a large difference. More likely, there is likely drug interaction between metformin and rapamycin that in some way impairs absorption or metabolism of these compounds. While these drugs have not been reported to interact directly, there are several drug interactions known to alter metabolism of metformin [43]. Importantly, the final concentrations of metformin and rapamycin even in combination are within the range of effective concentrations clinically. In diabetic patients prescribed metformin as part of their treatment, clinically relevant plasma concentrations of metformin are reported to be in the range of 10 µM, or approximately 1.3 mg/mL [44].

In contrast to previous studies, we found no evidence that metformin inhibited mTOR signaling when given as a single therapeutic [30]. Interestingly, we did find that metformin appeared to impair the ability of rapamycin to inhibit mTOR in the liver when both drugs were given concurrently. It is not clear why only this tissue, and this sex, seemed to show this effect. However, this could partially explain why female, but not male, mice benefitted metabolically from metformin. It would be of interest to further test whether metformin could alleviate the gluconeogenesis defect previously shown in some mTOR mutant mice [18]. This lack of effect on mTOR signaling may also partially explain why metformin at this dose failed to extend longevity in a similar cohort of HET3 mice [32]. One interpretation might be that metformin has no, or only mild, effects on physiological markers such as metabolism or longevity unless under a mildly stressful conditions, i.e., diabetes or metabolic dysfunction caused by rapamycin. Under this scenario, metformin may be more beneficial to aging and longevity in the human condition where there are no strict dietary and environmental controls as found in the animal vivarium. Certainly upcoming human studies could be used to address this type of question [45]. However, our study here shows that metformin is certainly valuable as part of a combined treatment with rapamycin and overall contributes to growing sets of data that alternative treatment regimens with rapamycin may be used to maximize effects on longevity and minimize side-effects [10,13,14,32,46].

## METHODS

### Mice and diets

Details of breeding and husbandry have been described elsewhere [1,2,37]. Males and females were separated at weaning and housed at a cage density of five mice per cage. All experiments were approved by the Institutional Animal Care and Use Committees at UTHSCSA and were performed under the supervision of the animal core facility of the Barshop Institute at UTHSCSA. At four months of age, mice were fed either LabDiet 5LG6 (PMI Nutrition International, Bentwood, MO, USA) diet alone or this diet containing either rapamycin (14 ppm), metformin (0.1% or 1000 ppm) or a combination of both rapamycin and metformin at these doses. Further discussion of diet formulations are discussed in [32].

### Assessment of glucose metabolism

Glucose and insulin tolerance tests were performed by fasting the mice for 6 hours (8:00-14:00) and then injecting either glucose (1.5 g kg^-1^) or insulin (0.75U kg^-1^) intraperitoneally as previously described [47]. Glucose measurements were performed at the specific time points indicated using a handheld OneTouch Ultra glucometer (LifeScan, Inc., Milpitas, CA) and test strips. Glucose and insulin tolerance tests were repeated in mice at 1, 2, 3 and 9 months after treatment was initiated. Areas under curve were calculated using the trapezoidal method from absolute glucose data determining during each tolerance test. Mice were euthanized after 9 months of treatment and tissues collected, processed and frozen at -80° C for future use. All mice were euthanized in the morning (09:00-11:00) and were given ad libitum access to food and water prior to sacrifice. Insulin (Crystal Chem, Downer’s Grove IL), triglycerides (Sigma, St. Louis MO), adiponectin and leptin (Millipore, Billerica MA) kits were used to measure metabolite concentrations in plasma per manufacturer’s instructions. Hepatic pyruvate and lactate were measured using commercial assay kits (Cayman Chemicals, Ann Arbor MI).

### Immunoblot

Total protein extracts from skeletal muscle, adipose and liver were created by homogenization of frozen tissue samples in RIPA buffer with additional protease and phosphatase inhibitors (Thermo Scientific, Rockford IL), followed by centrifuged at 14,000g at 4°C for 15 minutes. Protein content of the resultant supernatant was measured by BCA assay and equal amounts of protein samples were separated by SDS-PAGE, transferred to polyvinylidene difluoride membrane (Millipore) and subjected to immunoblot using the following antibodies and catalog numbers (Cell Signaling, Beverely MA): phosphorylated (Ser 235/236; #CS4857) and total ribosomal protein S6 (#CS2217), phosphorylated (Ser473; #CS9731) and total Akt (#CS9272), phosphorylated AMPKα (Thr172; #CS2535) and total AMPKα (#CS5831), and phosphorylated Acetyl-CoA carboxylase (Ser79; #CS11818) and total ACC (#CS3676). Protein bands were visualized using alkaline phosphatase-conjugated secondary antibodies (Santa Cruz Biotechnology, Santa Cruz CA) and ECL reagent. All immunoblots were quantified using ImageJ (NIH).

### RT-PCR

Total RNA was prepared from liver using TriReagent (Sigma) which was then treated with DNase I (Invitrogen, Carlsbad CA). cDNA was generated using Retroscript for RT-PCR (ThermoFisher, Waltham MA). Primers for PEPCK: (F) 5’-3’:TCTCTGATCCAGACCTTCCAA; (R) 5’-3’: GAAGTCCAGACCGTTATGCAG. Primers for G6P: (F) 5’-3’: GAAGGCCAAGAGATGGTGTGA; (R) 5’-3’:TGCAGCTCTTGCGGTACATG. Expression of actin as a housekeeping gene was used for normalization. Real-time PCR was performed in Applied Biosystems 7900 Real-Time PCR system with default PCR program. Triplicate samples were run in in 384-well real time PCR plate using a final volume of 10 µl with 16.26 µl SYBR Green PCR Master Mix (Applied Biosystems, Waltham MA), 160 nM forward primer, 160 nM reverse primer, and 8 ng template cDNA in each well. Ct values generated from these PCR were analyzed by relative quantification to values generated for control mice of each sex.

### Rapamycin blood concentrations

Whole blood was collected at sacrifice and analyzed for rapamycin and metformin concentrations by the San Antonio Nathan Shock Center Bioanalytical Pharmacology Core [1,48].

### Statistical analysis

Male and female data were analyzed separately by 2 way ANOVA using rapamycin and metformin treatment as the two subject factors. Male and female data were analyzed separately due to well-known sex difference in both the basic physiology of this strain of mice (HET3) as well as the difference in effect of rapamycin published elsewhere. Post-hoc analyses were performed using the method of Holm-Sidak. Where applicable, Student t-tests were used to compare 2 groups of interest. Statistical significance was attributed to all data for which a p value of less than or equal to 0.05.

## Supplementary Material

Supplementary File
