# Supplementary material for: Metformin reduces glucose intolerance caused by rapamycin treatment in genetically heterogeneous female mice
Source: Aging (Albany NY). 2018 Mar 22;10(3):386–401. doi: 10.18632/aging.101401 (PMC5892694; doi:10.18632/aging.101401)
Supplement: Supplementary File [file aging-10-101401-s001.pdf]

SUPPLEMENTARY MATERIAL

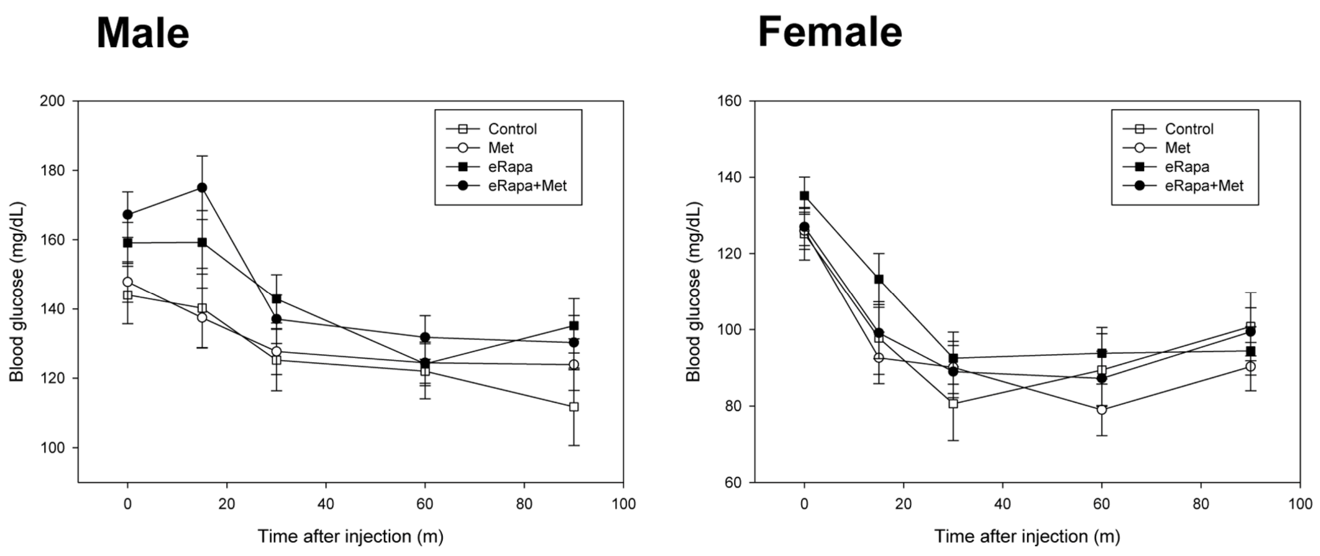

Supplementary Figure 1.

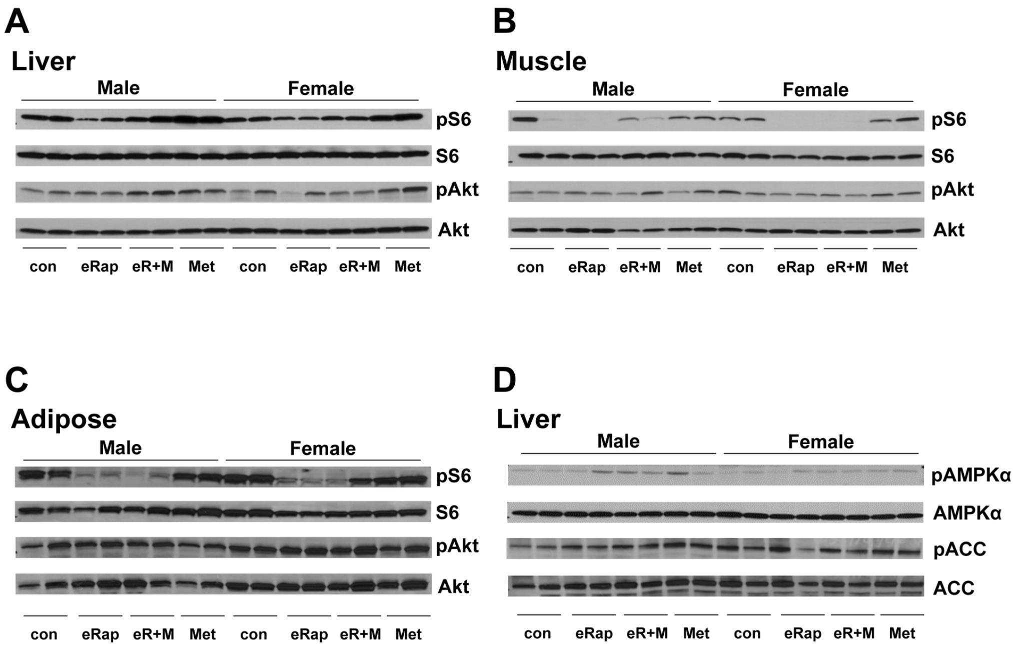

Supplementary Figure 2.

Supplementary Table 1. Tissue masses collected after 9 months of indicated treatment.

	Control	Met	eRapa	eRapa+Met	(p) eRapa	(p) Met	(p) Interact
Males							
Epi fat	1.53 (0.14)	1.34 (0.13)	0.85 (0.14)	1.19 (0.13)	***0.004***	0.59	0.057
Peri fat	0.70 (0.07)	0.59 (0.07)	0.36 (0.08)	0.45 (0.07)	***0.002***	0.88	0.18
Subc fat	1.09 (0.13)	0.85 (0.12)	0.54 (0.14)	0.96 (0.12)	0.09	0.44	***0.015***
Brown fat	0.29 (0.02)	0.26 (0.02)	0.20 (0.02)	0.23 (0.02)	***0.018***	0.85	0.52
Liver	1.79 (0.13)	1.81 (0.12)	1.77 (0.14)	2.07 (0.12)	0.34	0.22	0.26
Spleen	0.09 (0.00)	0.10 (0.00)	0.10 (0.00)	0.11 (0.00)	0.19	0.13	0.71
Pancreas	0.24 (0.03)	0.24 (0.02)	0.30 (0.03)	0.27 (0.02)	0.07	0.51	0.43
Kidney	0.55 (0.03)	0.60 (0.03)	0.60 (0.03)	0.67 (0.03)	0.07	0.06	0.75
Heart	0.18 (0.03)	0.21 (0.02)	0.23 (0.02)	0.23 (0.02)	0.08	0.55	0.46
Gastroc	0.40 (0.02)	0.37 (0.02)	0.34 (0.02)	0.36 (0.02)	***0.017***	0.70	0.14
Females							
Epi fat	3.27 (0.67)	2.47 (0.44)	2.10 (0.99)	2.76 (0.45)	0.37	0.88	0.14
Peri fat	1.34 (0.18)	1.10 (0.17)	0.78 (0.12)	0.86 (0.16)	***0.017***	0.63	0.31
Subc fat	0.81 (0.09)	0.80 (0.60)	0.55 (0.07)	0.70 (0.09)	***0.028***	0.37	0.29
Brown fat	0.22 (0.03)	0.18 (0.03)	0.15 (0.03)	0.15 (0.03)	0.07	0.54	0.57
Liver	1.46 (0.07)	1.44 (0.09)	1.38 (0.07)	1.34 (0.07)	0.23	0.67	0.87
Spleen	0.09 (0.01)	0.13 (0.01)	0.10 (0.01)	0.10 (0.01)	0.44	0.19	0.10
Pancreas	0.20 (0.02)	0.24 (0.02)	0.26 (0.02)	0.23 (0.02)	0.11	0.88	0.52
Kidney	0.41 (0.02)	0.38 (0.02)	0.32 (0.01)	0.33 (0.01)	***<0.001***	0.65	0.35
Heart	0.16 (0.02)	0.16 (0.01)	0.14 (0.01)	0.16 (0.01)	0.21	0.47	0.40
Gastroc	0.37 (0.03)	0.30 (0.01)	0.31 (0.03)	0.30 (0.01)	0.15	***0.04***	0.09

Epi fat = epigonadal fat, Peri fat = perirenal fat, Subc fat = subcutaneous fat, Gastroc = gastrocnemius. Masses are all given in g (± standard error of the mean). P values given are calculated from 2 way ANOVA. Those in bold and italicized reach statistical significance as defined by p < 0.05. For all groups, n = 8-10.

Supplementary Table 2. 2 Way ANOVA on glucose tolerance (AUC) each time point.

Sex	Time on diet	(F,p) eRapa	(F,p) Met	(F,p) Interact
Male	1 mo	***(43.2, <0.001)***	(1.9, 0.17)	(0.0, 0.85)
	2 mo	***(67.6, <0.001)***	(0.0, 0.91)	(0.1, 0.81)
	3 mo	***(67.5, <0.001)***	(0.0, 0.85)	(0.7, 0.41)
	9 mo	***(91.3, <0.001)***	*(3.2, 0.058)*	(1.2, 0.27)
Female	1 mo	***(15.0, <0.001)***	(0.7, 0.41)	(0.2, 0.63)
	2 mo	***(9.7, 0.004)***	(0.7, 0.42)	(0.2, 0.67)
	3 mo	***(5.6, 0.024)***	(2.5, 0.12)	(0.6, 0.43)
	9 mo	***(17.4, <0.001)***	***(7.5, 0.011)***	*(3.0, 0.09)*

P values given are calculated from two way ANOVA. Those in bold and italicized reach statistical significance as defined by p < 0.05. For all, group size is 8-10.

Supplementary Table 3. Fasting blood glucose concentrations during intervention study.

Time on diet	Control	Met	eRapa	eRapa+Met	(p) eRapa	(p) Met	(p) Interact
Males							
1 mo	156.6 (2.3)	154.8 (5.8)	165.6 (3.9)	172.1 (4.6)	***<0.001***	***0.038***	0.98
2 mo	146.4 (6.9)	133.5 (6.7)	161.9 (7.6)	156.3 (2.8)	***0.007***	0.17	0.58
3 mo	137.0 (9.6)	144.1 (5.4)	172.7 (3.9)	169.7 (3.4)	***<0.001***	0.70	0.35
9 mo	124.0 (15.0)	120.2 (8.5)	175.1 (10.1)	213.5 (10.5)	***<0.001***	0.16	0.09
Females							
1 mo	130.6 (4.7)	115.3 (4.2)	130.1 (3.4)	126.0 (4.2)	0.23	***0.025***	0.18
2 mo	125.8 (6.4)	122.6 (5.1)	135.2 (5.7)	132.0 (5.6)	0.15	0.62	0.99
3 mo	128.6 (5.6)	130.6 (3.4)	131.4 (4.7)	132.2 (4.9)	0.65	0.77	0.90
9 mo	122.2 (2.3)	108.3 (4.8)	127.5 (4.0)	116.1 (3.2)	0.13	***0.006***	0.77

Fasting blood glucose concentration after 6 hour fast (08:00-14:00). Data given as mean (± SEM) concentration in mg/dL for each group. P values given are calculated from two way ANOVA. Those in bold and italicized reach statistical significance as defined by p < 0.05. For all groups, n=10.

Supplementary Table 4. 2 Way ANOVA analyses on tissue mTORC1 (p-S6/total S6) signaling.

Sex	Tissue	(F,p) eRapa	(F,p) Met	(F,p) Interact
Male	Liver	***(11.1, 0.006)***	(1.5, 0.24)	(0.1, 0.73)
	Muscle	***(15.8, 0.002)***	(0.2, 0.64)	(0.7, 0.41)
	Adipose	***(4.2, 0.05)***	(0.0, 0.90)	(0.2, 0.69)
Female	Liver	(1.5, 0.24)	(1.5, 0.24)	(0.5, 0.51)
	Muscle	***(9.4, 0.01)***	(0.3, 0.62)	(0.0, 0.85)
	Adipose	***(5.6, 0.035)***	(0.1, 0.81)	(0.2, 0.69)

P values given are calculated from two way ANOVA. Those in bold and italicized reach statistical significance as defined by p < 0.05. For all, group size is 6.

Supplementary Table 5. 2 Way ANOVA analyses on tissue mTORC2 (p-Akt/total Akt) signaling.

Sex	Tissue	(F,p) eRapa	(F,p) Met	(F,p) Interact
Male	Liver	***(11.1, 0.006)***	(1.5, 0.24)	(0.1, 0.73)
	Muscle	***(15.8, 0.002)***	(0.2, 0.64)	(0.7, 0.41)
	Adipose	***(4.2, 0.05)***	(0.0, 0.90)	(0.2, 0.69)
Female	Liver	(1.5, 0.24)	(1.5, 0.24)	(0.5, 0.51)
	Muscle	***(9.4, 0.01)***	(0.3, 0.62)	(0.0, 0.85)
	Adipose	***(5.6, 0.035)***	(0.1, 0.81)	(0.2, 0.69)

P values given are calculated from two way ANOVA. Those in bold and italicized reach statistical significance as defined by p < 0.05. For all, group size is 6.
